# *N*-Methyl-l-leucyl-l-leucine hydro­chloride monohydrate

**DOI:** 10.1107/S1600536811031126

**Published:** 2011-08-27

**Authors:** Tao Lu, Mu-Wu Xu, Xiao-Jian Liao, Shi-Hai Xu

**Affiliations:** a Department of Chemistry, Jinan University, Guangzhou 510632, People’s Republic of China; b Guangdong Guangya High School, Guangdong 510160, People’s Republic of China

## Abstract

In the title compound C_13_H_27_N_2_O_3_^+^·Cl^−^·H_2_O, obtained by deprotecting the amino and carboxyl groups of an inter­mediate in the synthesis of the cyclic penta­peptide Galaxamide, a number of hydrogen-bonding inter­actions occur including aminium N—H⋯Cl, amide–carboxyl N—H⋯O, water O—H⋯Cl and carbox­yl–water O—H⋯O associations. The aminium N—H⋯Cl⋯H—N bridging extensions give rise to zigzag chains extending along the *a* axis, the overall two-dimensional structure lying in the (110) plane.

## Related literature

For general background to peptides, see: Humphrey & Chamberlin (1997 ▶). For the synthesis of Galaxamide, see: Xu, Liao, Xu *et al.* (2008 ▶); Rodriguez *et al.* (2007 ▶). For related structures, see: Liao *et al.* (2007 ▶); Xu, Liao, Diao *et al.* (2008 ▶). 
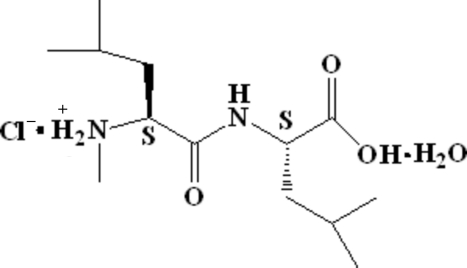


## Experimental

### 

#### Crystal data

C_13_H_27_N_2_O_3_^+^·Cl^−^·H_2_O
*M* *_r_* = 312.83
Monoclinic, 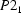

*a* = 5.2212 (2) Å
*b* = 9.6032 (5) Å
*c* = 18.4081 (8) Å
β = 96.329 (4)°
*V* = 917.36 (7) Å^3^
*Z* = 2
Mo *K*α radiation
μ = 0.22 mm^−1^
*T* = 295 K
0.45 × 0.32 × 0.17 mm

#### Data collection

Oxford Diffraction Xcalibur Sapphire3 Gemini Ultra CCD diffractometer
Absorption correction: multi-scan (*CrysAlis PRO*; Agilent, 2011 ▶) *T*_min_ = 0.990, *T*_max_ = 1.000
3703 measured reflections
2616 independent reflections
2271 reflections with *I* > 2s*I*)
*R*_int_ = 0.015

#### Refinement

*R*[*F* ^2^ > 2σ(*F* ^2^)] = 0.042
*wR*(*F* ^2^) = 0.114
*S* = 1.01
2616 reflections
186 parameters
1 restraint
H-atom parameters constrained
Δρ_max_ = 0.37 e Å^−3^
Δρ_min_ = −0.22 e Å^−3^
Absolute structure: Flack (1983 ▶), 686 Friedel pairs
Flack parameter: −0.01 (8)

### 

Data collection: *CrysAlis PRO* (Agilent, 2011 ▶); cell refinement: *CrysAlis PRO*; data reduction: *CrysAlis PRO*; program(s) used to solve structure: *SHELXS97* (Sheldrick, 2008 ▶); program(s) used to refine structure: *SHELXL97* (Sheldrick, 2008 ▶); molecular graphics: *OLEX2* (Dolomanov *et al.*, 2009 ▶); software used to prepare material for publication: *OLEX2*.

## Supplementary Material

Crystal structure: contains datablock(s) I, global. DOI: 10.1107/S1600536811031126/zs2126sup1.cif

Structure factors: contains datablock(s) I. DOI: 10.1107/S1600536811031126/zs2126Isup2.hkl

Supplementary material file. DOI: 10.1107/S1600536811031126/zs2126Isup3.cml

Additional supplementary materials:  crystallographic information; 3D view; checkCIF report

## Figures and Tables

**Table 1:** Hydrogen-bond geometry (Å, °)

*D*—H⋯*A*	*D*—H	H⋯*A*	*D*⋯*A*	*D*—H⋯*A*
N1—H1*A*⋯Cl1^i^	0.90	2.31	3.174 (2)	161
N1—H1*B*⋯Cl1	0.90	2.25	3.092 (2)	155
N2—H2⋯O2^ii^	0.86	2.40	3.006 (3)	128
O3—H3*A*⋯O4	0.85	1.74	2.591 (5)	179
O4—H4*A*⋯Cl1^iii^	0.85	2.51	3.198 (4)	139
O4—H4*B*⋯Cl1^iv^	0.85	2.48	3.182 (4)	141

Symmetry codes: (i) 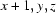; (ii) 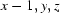; (iii) 
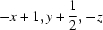
; (iv) 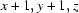.

**Fig. 1.**
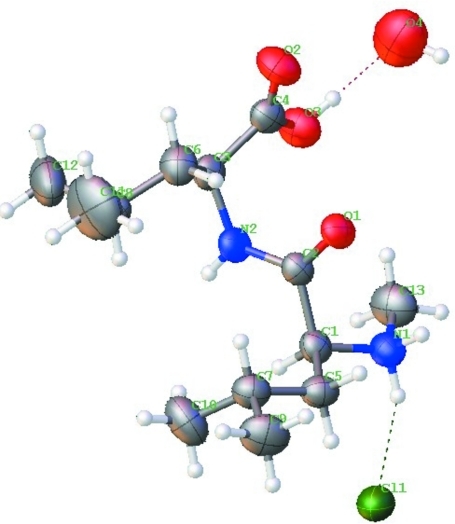
The molecular structure of the title compound showing the atom numbering scheme. Inter-species hydrogen bonds are shown as dashed lines and displacement ellipsoids are drawn at the 50% probability level.

**Fig. 2.**
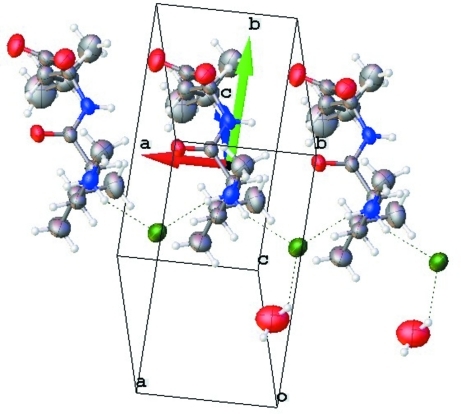
Fgure 2. A perspective view of the packing in the unit cell showing the hydrogen-bonding interactions as dashed lines.

## supplementary crystallographic information

### Comment

Peptide compounds play an important role in life activities (Humphrey & Chamberlin, 1997). The title compound C_13_H_27_N_2_O_3_^+^ Cl^-^ . H_2_O (Fig. 1) is a modified dipeptide employed in the synthesis of the cytotoxic cyclic pentapeptide Galaxamide (Xu, Liao, Xu *et al.*, 2008), obtained by deprotecting the amino and carboxyl groups of the intermediate (Rodriguez *et al.*, 2007). The purpose was to explore the activity targets of the intermediates in relation to those of the target compound (Liao *et al.*, 2007, Xu, Liao, Diao *et al.*, 2008). In the crystal structure of the title compound, there are a number of intermolecular hydrogen-bonding interactions (Table 1), including aminium N—H···Cl, amide N—H···O_carboxyl_, water O—H···Cl and carboxylic acid O—H···O_water_ associations. The aminium N—H···Cl···H—N bridging extensions give zigzag chains extending along the *a* axis in the unit cell, the overall two-dimensional structure lying along (110) (Fig 2).

### Experimental

Diisopropylethylamine (DIPEA) (6 mmol, 1.1 ml) was added dropwise to a stirred solution of *L*-leucine benzyl ester *p*-toluenesulfonate (6 mmol, 2.36 g) in anhydrous THF (8 ml) at 273 K under nitrogen and stirred for 15 min. The coupling reagent DEPBT (6 mmol, 1.8 g) was added to a stirred solution of N-Boc-Me—*L*-Leu-OH (5 mmol, 1.30 g) in anhydrous THF (5 ml) at 273 K under nitrogen and the suspension was stirred for 15 min. A suspension of *L*-leucine benzyl ester *p*-toluenesulfonate was added by cannula to the N-Boc-Me—*L*-Leu-OH suspension at 273 K under nitrogen and the mixture was allowed to warm to room temperature over the course of 24 h, then evaporated *in vacuo*. The crude product was then purified by chromatography on silica using *n*-hexane/acetone (20:1) as eluent to give the dipeptide as colorless crystals (yield 2.1g: 92.5%). This dipeptide (4 mmol, 1.8 g) was dissolved in CH_2_Cl_2_ (7 ml) and 2 ml of TFA was added dropwise at 273 K under nitrogen using a constant pressure funnel. The mixture was stirred at 273 K until the starting material disappeared (monitored by TLC). The solution was concentrated *in vacuo*, the residue was dissolved in CH_2_Cl_2_2 and concentrated again to remove the Boc dipeptide derivative which was dried *in vacuo*. This Boc derivative (3 mmol, 1.91 g) was reduced with hydrogen (0.1 Mpa) and 10% Pd—C (0.62 g) in ethyl acetate (40 ml) until the starting material disappeared (monitored using TLC). The Pd—C was filtered, and the filtrate was concentrated *in vacuo* to obtain the title compound  (yield 1.85 g: 97%). Colourless crystals suitable for X-ray analysis grew over a period of a week from a solution in methanol containing a small amount of dilute HCl, when exposed to air.

### Refinement

The C-bound and O-bound H atoms were positioned geometrically and were included in the refinement in the riding-model approximation, with distances 0.96 Å (CH_3_), 0.97 Å (CH_2_), 0.98 (CH), or 0.85 Å (OH) and U_iso_(H) = 1.2U_eq_(C, O) for methine, methylene, hydroxyl and carboxyl H atoms, and U_iso_ = 1.5U_eq_(C) for methyl H atoms. The N H-atoms were located in a difference-Fourier synthesis and then refined as riding on the N atoms with U_iso_(H) = 1.2U_eq_(N). The known S absolute configuration for L-leucine [(S)-2-amino-4-methylvaleric acid] was invoked for both chiral centres in the title molecule (C1S,C3S).

### Crystal data

**Table:**

C_13_H_27_N_2_O_3_^+^·Cl^−^·H_2_O	*F*(000) = 340
*M**_r_* = 312.83	*D*_x_ = 1.133 Mg m^−^^3^
Monoclinic, *P*2_1_	Mo *K*α radiation, λ = 0.7107 Å
*a* = 5.2212 (2) Å	Cell parameters from 1351 reflections
*b* = 9.6032 (5) Å	θ = 3.1–29.1°
*c* = 18.4081 (8) Å	µ = 0.22 mm^−^^1^
β = 96.329 (4)°	*T* = 295 K
*V* = 917.36 (7) Å^3^	Block, colourless
*Z* = 2	0.45 × 0.32 × 0.17 mm

### Data collection

**Table:**

Oxford Diffraction Xcalibur Sapphire3 Gemini Ultra CCD diffractometer	2616 independent reflections
Radiation source: Enhance (Mo) X-ray Source	2271 reflections with *I* > 2s˘*I*)
graphite	*R*_int_ = 0.015
Detector resolution: 16.0288 pixels mm^-1^	θ_max_ = 26.0°, θ_min_ = 3.1°
ω scans	*h* = −6→6
Absorption correction: multi-scan (*CrysAlis PRO*; Agilent, 2011)	*k* = −7→11
*T*_min_ = 0.990, *T*_max_ = 1.000	*l* = −19→22
3703 measured reflections	

### Refinement

**Table:**

Refinement on *F*^2^	Secondary atom site location: difference Fourier map
Least-squares matrix: full	Hydrogen site location: inferred from neighbouring sites
*R*[*F*^2^ > 2σ(*F*^2^)] = 0.042	H-atom parameters constrained
*wR*(*F*^2^) = 0.114	*w* = 1/[σ^2^(*F*_o_^2^) + (0.065*P*)^2^ + 0.1023*P*] where *P* = (*F*_o_^2^ + 2*F*_c_^2^)/3
*S* = 1.01	(Δ/σ)_max_ = 0.001
2616 reflections	Δρ_max_ = 0.37 e Å^−^^3^
186 parameters	Δρ_min_ = −0.22 e Å^−^^3^
1 restraint	Absolute structure: Flack (1983), 686 Friedel pairs
Primary atom site location: structure-invariant direct methods	Flack parameter: −0.01 (8)

### Special details

**Table:**

Geometry. Bond distances, angles etc. have been calculated using the rounded fractional coordinates. All su's are estimated from the variances of the (full) variance-covariance matrix. The cell esds are taken into account in the estimation of distances, angles and torsion angles
Refinement. Refinement of F^2^ against ALL reflections. The weighted R-factor wR and goodness of fit S are based on F^2^, conventional R-factors R are based on F, with F set to zero for negative F^2^. The threshold expression of F^2^ > 2sigma(F^2^) is used only for calculating R-factors(gt) etc. and is not relevant to the choice of reflections for refinement. R-factors based on F^2^ are statistically about twice as large as those based on F, and R- factors based on ALL data will be even larger.

### Fractional atomic coordinates and isotropic or equivalent isotropic displacement parameters (Å^2^)

**Table:**

	*x*	*y*	*z*	*U*_iso_*/*U*_eq_	
O1	0.9159 (3)	0.8611 (2)	0.21026 (11)	0.0456 (7)	
O2	1.1446 (4)	1.1632 (3)	0.27116 (12)	0.0647 (9)	
O3	0.8114 (4)	1.1739 (3)	0.18571 (12)	0.0609 (8)	
N1	0.5270 (4)	0.7595 (3)	0.09916 (12)	0.0467 (8)	
N2	0.5803 (4)	0.9650 (3)	0.25512 (13)	0.0422 (8)	
C1	0.4995 (5)	0.7609 (3)	0.17896 (15)	0.0407 (9)	
C2	0.6837 (5)	0.8673 (3)	0.21552 (14)	0.0376 (8)	
C3	0.7383 (5)	1.0686 (3)	0.29656 (15)	0.0429 (9)	
C4	0.9252 (5)	1.1384 (3)	0.24936 (15)	0.0451 (9)	
C5	0.5595 (5)	0.6146 (4)	0.20897 (15)	0.0460 (8)	
C6	0.8781 (6)	1.0087 (4)	0.36714 (16)	0.0538 (10)	
C7	0.5536 (5)	0.5991 (4)	0.29136 (15)	0.0477 (9)	
C8	0.7036 (7)	0.9642 (5)	0.42373 (18)	0.0648 (13)	
C9	0.6292 (8)	0.4502 (4)	0.3139 (2)	0.0718 (14)	
C10	0.2924 (7)	0.6351 (6)	0.3153 (2)	0.0761 (14)	
C11	0.8619 (12)	0.8836 (9)	0.4839 (3)	0.124 (3)	
C12	0.5709 (8)	1.0854 (7)	0.4543 (2)	0.0883 (19)	
C13	0.4878 (8)	0.8963 (5)	0.06236 (19)	0.0701 (14)	
O4	1.0969 (8)	1.2934 (4)	0.09741 (19)	0.1200 (16)	
Cl1	0.01614 (13)	0.60884 (9)	0.04716 (4)	0.0580 (3)	
H1	0.32240	0.78610	0.18640	0.0490*	
H1A	0.68570	0.72850	0.09280	0.0560*	
H1B	0.41260	0.69860	0.07710	0.0560*	
H2	0.41610	0.96640	0.25610	0.0510*	
H3	0.62160	1.14120	0.31070	0.0520*	
H3A	0.90570	1.21190	0.15680	0.0730*	
H5A	0.43590	0.55010	0.18430	0.0550*	
H5B	0.72900	0.58780	0.19700	0.0550*	
H6A	0.97830	0.92880	0.35480	0.0640*	
H6B	0.99780	1.07820	0.38890	0.0640*	
H7	0.68190	0.66260	0.31610	0.0570*	
H8	0.57160	0.90170	0.40000	0.0780*	
H9A	0.50160	0.38660	0.29190	0.1070*	
H9B	0.64010	0.44200	0.36620	0.1070*	
H9C	0.79340	0.42830	0.29790	0.1070*	
H10A	0.24780	0.72890	0.30110	0.1140*	
H10B	0.29980	0.62660	0.36750	0.1140*	
H10C	0.16460	0.57240	0.29260	0.1140*	
H11A	0.94290	0.80570	0.46310	0.1850*	
H11B	0.75120	0.85070	0.51860	0.1850*	
H11C	0.99160	0.94350	0.50820	0.1850*	
H12A	0.69730	1.14720	0.47860	0.1330*	
H12B	0.45900	1.05260	0.48870	0.1330*	
H12C	0.47140	1.13420	0.41540	0.1330*	
H13A	0.50050	0.88560	0.01100	0.1050*	
H13B	0.61720	0.96040	0.08280	0.1050*	
H13C	0.32020	0.93160	0.06940	0.1050*	
H4A	1.14740	1.23240	0.06880	0.1440*	
H4B	1.01050	1.35560	0.07270	0.1440*	

### Atomic displacement parameters (Å^2^)

**Table:**

	*U*^11^	*U*^22^	*U*^33^	*U*^12^	*U*^13^	*U*^23^
O1	0.0316 (9)	0.0457 (13)	0.0607 (12)	0.0060 (9)	0.0102 (8)	−0.0027 (11)
O2	0.0421 (11)	0.0755 (19)	0.0769 (14)	−0.0069 (12)	0.0078 (10)	0.0094 (14)
O3	0.0645 (13)	0.0608 (16)	0.0567 (12)	−0.0036 (13)	0.0038 (11)	0.0162 (12)
N1	0.0420 (12)	0.0530 (17)	0.0443 (13)	−0.0020 (12)	0.0011 (10)	0.0028 (12)
N2	0.0325 (11)	0.0422 (15)	0.0526 (13)	0.0089 (11)	0.0075 (10)	0.0002 (12)
C1	0.0340 (12)	0.0426 (17)	0.0462 (15)	0.0045 (13)	0.0077 (11)	0.0011 (14)
C2	0.0342 (12)	0.0355 (16)	0.0434 (14)	0.0058 (13)	0.0059 (11)	0.0057 (13)
C3	0.0397 (13)	0.0399 (18)	0.0499 (15)	0.0089 (13)	0.0081 (12)	−0.0017 (13)
C4	0.0446 (15)	0.0358 (18)	0.0555 (16)	0.0064 (14)	0.0083 (13)	0.0016 (14)
C5	0.0427 (13)	0.0393 (16)	0.0551 (15)	−0.0006 (15)	0.0019 (11)	0.0009 (16)
C6	0.0487 (16)	0.062 (2)	0.0497 (17)	0.0062 (16)	0.0009 (13)	0.0009 (16)
C7	0.0475 (14)	0.0412 (17)	0.0535 (15)	0.0020 (16)	0.0022 (12)	0.0052 (16)
C8	0.073 (2)	0.071 (3)	0.0502 (17)	−0.022 (2)	0.0056 (16)	0.0017 (18)
C9	0.076 (2)	0.050 (2)	0.088 (3)	0.004 (2)	0.003 (2)	0.018 (2)
C10	0.0628 (19)	0.092 (3)	0.077 (2)	0.008 (2)	0.0236 (17)	0.025 (3)
C11	0.151 (5)	0.144 (6)	0.077 (3)	0.009 (5)	0.018 (3)	0.054 (4)
C12	0.079 (2)	0.130 (5)	0.060 (2)	−0.009 (3)	0.0255 (18)	−0.015 (3)
C13	0.078 (2)	0.071 (3)	0.059 (2)	−0.003 (2)	−0.0021 (18)	0.018 (2)
O4	0.165 (3)	0.099 (3)	0.099 (2)	−0.003 (3)	0.028 (2)	0.015 (2)
Cl1	0.0507 (4)	0.0606 (5)	0.0630 (4)	−0.0075 (4)	0.0081 (3)	−0.0114 (5)

### Geometric parameters (Å, °)

**Table:**

O1—C2	1.228 (3)	C1—H1	0.9800
O2—C4	1.195 (3)	C3—H3	0.9800
O3—C4	1.300 (4)	C5—H5B	0.9700
O3—H3A	0.8500	C5—H5A	0.9700
O4—H4B	0.8500	C6—H6A	0.9700
O4—H4A	0.8500	C6—H6B	0.9700
N1—C13	1.482 (5)	C7—H7	0.9800
N1—C1	1.492 (4)	C8—H8	0.9800
N2—C2	1.338 (4)	C9—H9C	0.9600
N2—C3	1.454 (4)	C9—H9B	0.9600
N1—H1A	0.9000	C9—H9A	0.9600
N1—H1B	0.9000	C10—H10B	0.9600
N2—H2	0.8600	C10—H10A	0.9600
C1—C5	1.529 (5)	C10—H10C	0.9600
C1—C2	1.510 (4)	C11—H11B	0.9600
C3—C4	1.531 (4)	C11—H11A	0.9600
C3—C6	1.531 (4)	C11—H11C	0.9600
C5—C7	1.528 (4)	C12—H12B	0.9600
C6—C8	1.519 (5)	C12—H12C	0.9600
C7—C10	1.519 (5)	C12—H12A	0.9600
C7—C9	1.529 (5)	C13—H13C	0.9600
C8—C12	1.496 (7)	C13—H13A	0.9600
C8—C11	1.519 (8)	C13—H13B	0.9600
			
C4—O3—H3A	116.00	C3—C6—H6B	108.00
H4A—O4—H4B	110.00	C8—C6—H6A	109.00
C1—N1—C13	114.8 (3)	C3—C6—H6A	109.00
C2—N2—C3	121.7 (2)	H6A—C6—H6B	108.00
C1—N1—H1B	109.00	C8—C6—H6B	109.00
C13—N1—H1A	108.00	C5—C7—H7	108.00
H1A—N1—H1B	108.00	C9—C7—H7	108.00
C1—N1—H1A	109.00	C10—C7—H7	108.00
C13—N1—H1B	109.00	C6—C8—H8	108.00
C2—N2—H2	119.00	C11—C8—H8	108.00
C3—N2—H2	119.00	C12—C8—H8	108.00
N1—C1—C2	108.6 (2)	C7—C9—H9B	109.00
N1—C1—C5	108.0 (2)	C7—C9—H9C	110.00
C2—C1—C5	111.5 (2)	H9A—C9—H9B	109.00
O1—C2—C1	121.2 (2)	H9A—C9—H9C	109.00
N2—C2—C1	116.2 (2)	H9B—C9—H9C	109.00
O1—C2—N2	122.6 (3)	C7—C9—H9A	110.00
N2—C3—C6	112.2 (3)	C7—C10—H10A	109.00
C4—C3—C6	111.9 (2)	C7—C10—H10B	109.00
N2—C3—C4	111.2 (2)	H10A—C10—H10B	110.00
O2—C4—C3	123.0 (3)	H10A—C10—H10C	110.00
O3—C4—C3	111.7 (2)	H10B—C10—H10C	109.00
O2—C4—O3	125.2 (3)	C7—C10—H10C	110.00
C1—C5—C7	115.0 (3)	C8—C11—H11B	109.00
C3—C6—C8	115.0 (3)	C8—C11—H11C	109.00
C5—C7—C10	112.5 (2)	C8—C11—H11A	110.00
C9—C7—C10	110.3 (3)	H11A—C11—H11C	109.00
C5—C7—C9	109.1 (3)	H11B—C11—H11C	109.00
C6—C8—C12	112.1 (4)	H11A—C11—H11B	109.00
C11—C8—C12	111.1 (4)	C8—C12—H12A	109.00
C6—C8—C11	108.9 (3)	C8—C12—H12B	109.00
C2—C1—H1	110.00	H12A—C12—H12B	109.00
C5—C1—H1	110.00	H12A—C12—H12C	109.00
N1—C1—H1	110.00	C8—C12—H12C	110.00
C4—C3—H3	107.00	H12B—C12—H12C	109.00
C6—C3—H3	107.00	N1—C13—H13B	109.00
N2—C3—H3	107.00	N1—C13—H13C	109.00
C1—C5—H5B	109.00	N1—C13—H13A	110.00
C7—C5—H5A	108.00	H13A—C13—H13C	109.00
C7—C5—H5B	109.00	H13B—C13—H13C	109.00
H5A—C5—H5B	107.00	H13A—C13—H13B	109.00
C1—C5—H5A	109.00		
			
C13—N1—C1—C2	−56.0 (3)	C2—C1—C5—C7	57.8 (3)
C13—N1—C1—C5	−177.1 (2)	N2—C3—C4—O2	−138.2 (3)
C3—N2—C2—O1	−1.8 (4)	N2—C3—C4—O3	45.8 (3)
C3—N2—C2—C1	176.3 (2)	C6—C3—C4—O2	−11.8 (4)
C2—N2—C3—C4	49.6 (3)	C6—C3—C4—O3	172.1 (3)
C2—N2—C3—C6	−76.5 (3)	N2—C3—C6—C8	−66.0 (4)
N1—C1—C2—O1	−56.6 (3)	C4—C3—C6—C8	168.1 (3)
N1—C1—C2—N2	125.2 (3)	C1—C5—C7—C9	−177.4 (3)
C5—C1—C2—O1	62.3 (3)	C1—C5—C7—C10	59.8 (4)
C5—C1—C2—N2	−115.9 (3)	C3—C6—C8—C11	169.8 (4)
N1—C1—C5—C7	177.1 (2)	C3—C6—C8—C12	−66.9 (4)

### Hydrogen-bond geometry (Å, °)

**Table:**

*D*—H···*A*	*D*—H	H···*A*	*D*···*A*	*D*—H···*A*
N1—H1A···Cl1^i^	0.90	2.31	3.174 (2)	161
N1—H1B···Cl1	0.90	2.25	3.092 (2)	155
N2—H2···O2^ii^	0.86	2.40	3.006 (3)	128
O3—H3A···O4	0.85	1.74	2.591 (5)	179
O4—H4A···Cl1^iii^	0.85	2.51	3.198 (4)	139
O4—H4B···Cl1^iv^	0.85	2.48	3.182 (4)	141
C1—H1···O1^ii^	0.98	2.33	3.306 (3)	175
C3—H3···O2^ii^	0.98	2.53	3.215 (3)	127

Symmetry codes: (i) *x*+1, *y*, *z*; (ii) *x*−1, *y*, *z*; (iii) −*x*+1, *y*+1/2, −*z*; (iv) *x*+1, *y*+1, *z*.
